# Transvaginal Needle Biopsy for the Diagnosis of Radiation-Induced Angiosarcoma in Cervical Cancer: Utility and Role of Next-Generation Sequencing

**DOI:** 10.7759/cureus.84869

**Published:** 2025-05-27

**Authors:** Yurina Shimomura, Michiko Kaneda, Naosuke Enomoto, Kenta Yoshida, Eiji Kondo

**Affiliations:** 1 Department of Obstetrics and Gynecology, Mie Central Medical Center, Tsu, JPN; 2 Department of Obstetrics and Gynecology, Mie University School of Medicine, Tsu, JPN

**Keywords:** cervical cancer, concurrent chemoradiotherapy, needle biopsy, next-generation sequencing, radiation-induced angiosarcoma

## Abstract

Concurrent chemoradiotherapy (CCRT) is a common treatment for advanced cervical cancer, and angiosarcoma rarely occurs at the irradiated site. We report a case of angiosarcoma diagnosed via a transvaginal ultrasound-guided needle biopsy of a mass in the rectovaginal space, which developed five years after CCRT in a patient with stage IIIB cervical cancer. The biopsy specimen was analyzed using next-generation sequencing (NGS), which identified relevant somatic genetic mutations. This case highlights the value of needle biopsy as a useful diagnostic tool and its potential role in guiding treatment decisions.

## Introduction

Concurrent chemoradiotherapy (CCRT) is commonly used as the primary treatment for advanced cervical cancer. Although CCRT is effective, it carries risks of late complications, one of which is radiation-induced sarcoma. Radiation-induced sarcomas account for less than 5% of all sarcomas and are associated with poor prognosis [1]. The diagnosis of radiation-induced sarcomas is defined as (1) history of previous radiotherapy, (2) occurrence of sarcoma within the radiation field, (3) more than a three-year latency period before sarcoma onset, and (4) pathologic confirmation of a sarcoma that was histologically different from primary cancer [2,3]. Radiation-induced angiosarcomas (RIAS) are even rarer, accounting for 15% of all radiation-induced sarcomas [1]. RIAS can occur throughout the body, but they are most commonly observed after radiation therapy for breast cancer [4]. The occurrence of RIAS in the gynecological region has rarely been reported, and treatment options are limited due to the rarity of the disease.

In this report, we present a case of RIAS in the pelvis, diagnosed after CCRT for cervical cancer, using transvaginal ultrasound-guided needle biopsy. We also discuss the utility of next-generation sequencing (NGS) analysis on biopsy specimens.

## Case presentation

**Figure 1:**
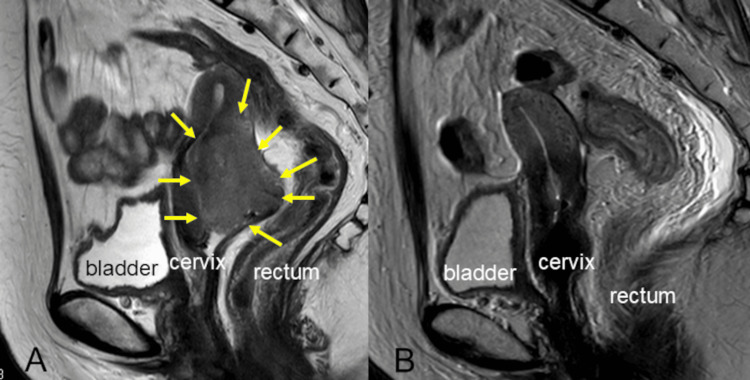
MRI images before and after primary treatment A: Before primary CCRT; B: After primary CCRT; Yellow arrow: cervical cancer CCRT: concurrent chemoradiotherapy

The patient was a 71-year-old woman with no personal or family history of cancer. She presented to the hospital with the chief complaint of irregular bleeding and was diagnosed with stage IIIB cervical cancer (International Federation of Gynecology and Obstetrics (FIGO) 2008), classified as cT1b3N0M0, with squamous cell carcinoma of the uterine cervix. Her serum squamous cell carcinoma (SCC) antigen level was elevated at 98.9 ng/mL. Magnetic resonance imaging (MRI) revealed a cervical tumor approximately 60 mm in size with bilateral parametrial invasion. The patient received pelvic external beam radiotherapy (EBRT) to a total dose of 50.4 Gy (1.8 Gy/fraction) along with vaginal brachytherapy, which was administered at 6 Gy per fraction to point A over 3 fractions. A right parametrium boost of 6 Gy was also delivered in 3 fractions. Chemotherapy consisted of 6 courses of weekly cisplatin (35 mg/m^2^, 53 mg/body). After completing treatment, the patient achieved a complete response and was followed up accordingly. MRI images before and after CCRT are shown in Figure 1.

**Figure 2:**
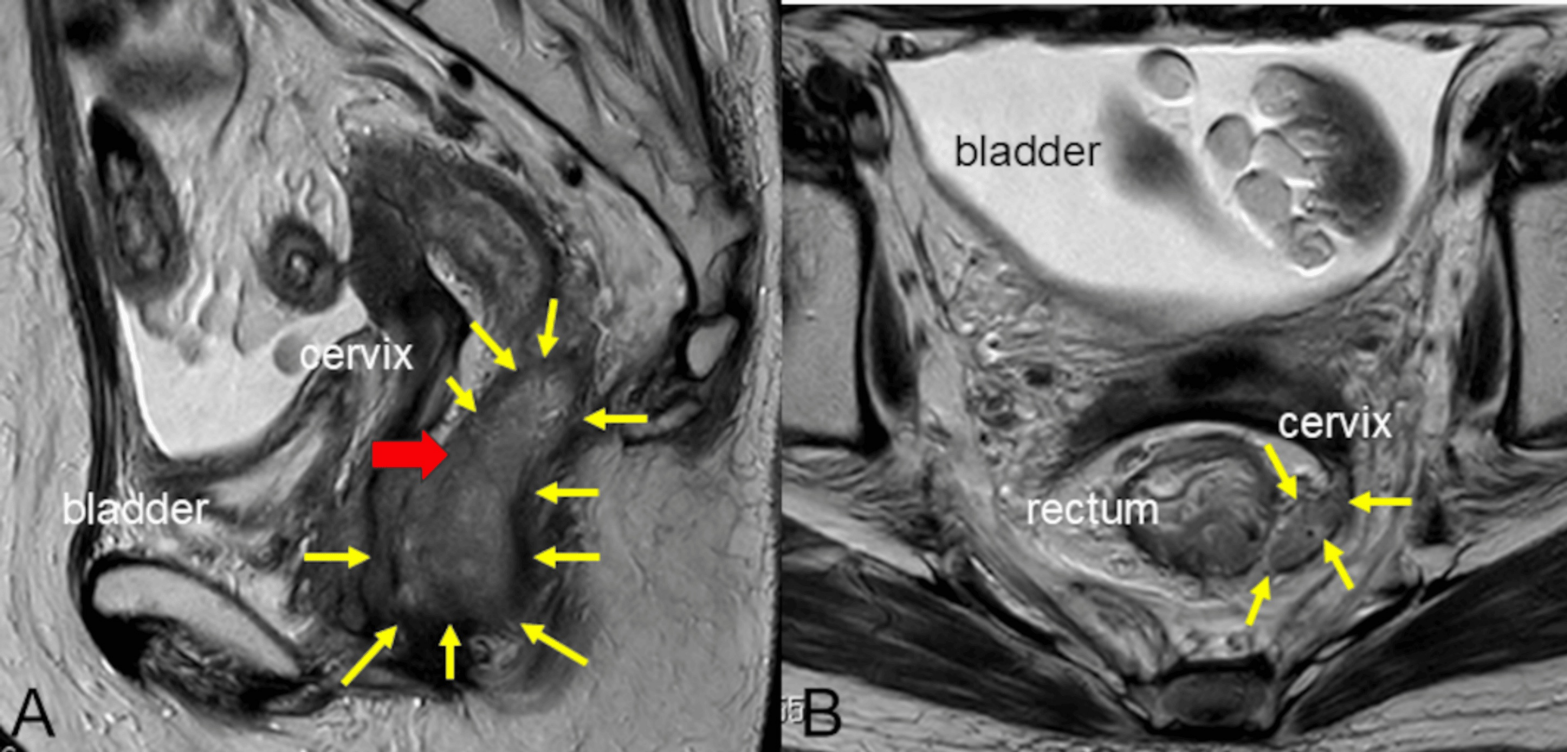
MRI images five years after CCRT A: Sagittal section; B: Horizontal section; Yellow arrow: tumor; Red arrow: biopsy point CCRT: concurrent chemoradiotherapy

Five years after the initial treatment, a computed tomography (CT) scan revealed a mass in the rectovaginal space, and recurrence was suspected. MRI showed a mass in the same area with a mildly high T2 signal and a high diffusion-weighted imaging DWI signal, suggestive of local recurrence (Figure 2). Additionally, lymph node metastasis was observed near the superior rectal artery. A positron emission tomography (PET)/CT scan showed F-18 fluorodeoxyglucose (FDG) accumulation with a standardized uptake value (SUV) of 17.37 in the vaginal to peri-rectal mass, SUV of 8.32 in the lymph node of the superior rectal artery, and SUV of 16.89 in the peritoneal dissemination of the liver surface. Vaginoscopy revealed no gross abnormalities in the cervix, and cervical cytology was negative for intraepithelial lesion or malignancy (NILM), with benign histology. Serum SCC and serum CEA levels were within normal limits (0.8 ng/mL and 2.1 ng/mL, respectively). Colonoscopy did not reveal any obvious neoplastic lesions.

**Figure 3:**
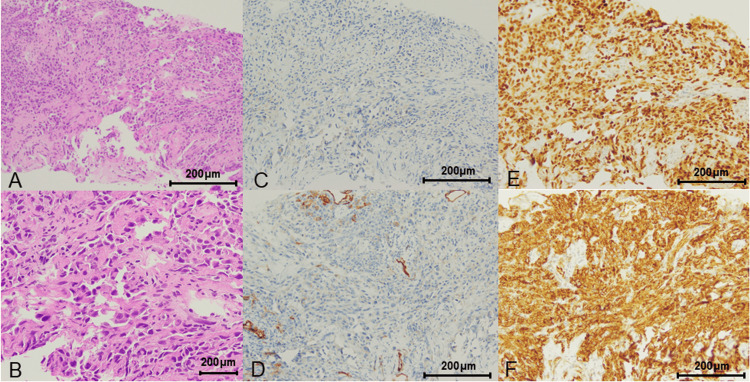
Histopathology of angiosarcoma Large nucleated atypical cells formed in the vascular lumens with infiltrative growth. A: Hematoxylin-eosin, original magnification x20; B: Hematoxylin-eosin, original magnification x40; C: Negative for pan cytokeratin AE1/AE3; D: Partially positive for CD 34; E: Strongly positive for ERG; F: Strongly positive for CD31

To establish a definitive pathological diagnosis, we performed transvaginal ultrasound-guided needle biopsy. Using a one-handed automated biopsy 18-gauge needle (PRIMECUT® II), we obtained tissue from three different sites of the pelvic tumor, with color Doppler used to identify vessels before puncture. Histopathological examination revealed large, atypical nucleated cells forming vascular lumens with infiltrative growth patterns. These cells were negative for cytokeratin CK AE1/3 and CK p40. However, vascular markers, including CD34 (partially positive), ERG (strongly positive), and CD31 (strongly positive), were diffusely expressed in the tumor cells (Figure 3). Based on these findings, the diagnosis of angiosarcoma was confirmed. The diagnosis of RIAS was established based on the criteria proposed by Cahan and Arlen [2,3].

**Figure 4:**
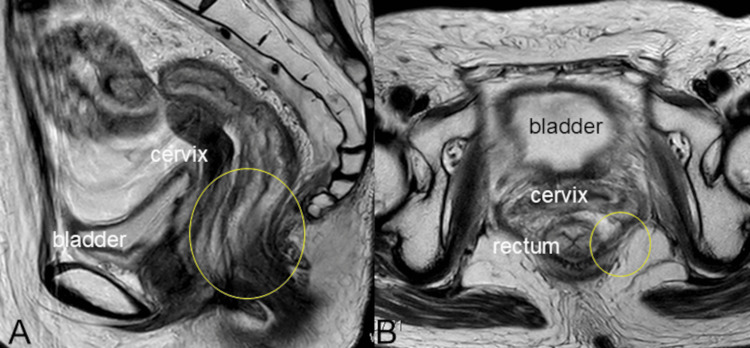
MRI images after weekly paclitaxel A Sagittal section of the MRI images after weekly paclitaxel; B Horizontal section of MRI images after weekly paclitaxel

The patient had peritoneal dissemination and was started on chemotherapy with weekly paclitaxel as the primary treatment. After four months of paclitaxel treatment, the patient achieved a complete response (Figure 4). However, during chemotherapy, the patient developed a cough, and a CT scan revealed interstitial pneumonia, which was treated with pulse steroid therapy.

NGS analysis of the biopsy specimen revealed a somatic *BRCA1 *gene mutation. The patient was eligible to participate in a clinical trial with olaparib in combination with pembrolizumab for homologous recombination repair mutations (HRRm) and/or homologous recombination deficiency (HRD) positive cancers (jRCT 2011200025), and a phase 2 study of niraparib in recurrent or persistent rare gynecologic malignancies with HRD (jRCT 2031210264). However, she was deemed unsuitable for these trials due to the interstitial pneumonia. Although paclitaxel was effective for approximately 16 months, the disease eventually progressed. She was switched to pazopanib, but disease progression occurred within 3 months, leading to death 35 months after the recurrence diagnosis.

## Discussion

RIAS can occur throughout the body, with the breast being the most common site. However, gynecological occurrences are extremely rare [5]. In a retrospective case-control study of angiosarcomas following radiotherapy for breast cancer, the incidence of breast/chest wall angiosarcoma was found to be 0.1% [6]. The median duration from radiotherapy to the development of angiosarcoma in breast cancer has been reported to be 8 years (range 3-20 years), whereas in cervical cancer, a range of 6-21 years is suggested [6].

The mechanism underlying RIAS involves DNA damage caused by radiation and the subsequent repair processes of various radiation-induced tissue damages [7]. Although the *BRCA1* DNA damage response pathway has been reported to be highly enriched in genetic variation in the NGS analyses of RIAS [7], further studies may be needed to corroborate the potential causal relationship between *BRCA1* mutation and RIAS development. In this case, NGS analysis also revealed a somatic *BRCA1* mutation. Radiation-induced angiosarcomas with *BRCA1* mutations are defective in the homologous recombination repair pathway and may be susceptible to platinum-based chemotherapy and poly ADP-ribose polymerase (PARP) inhibitors, and this case was also considered for clinical trials of PARP as a treatment option.

A systematic review of radiation-associated angiosarcoma of the breast reported a poor prognosis, with a 5-year overall survival rate of 43% [8], and the median survival after histological diagnosis ranged from 10.8 to 33.5 months [9]. For primary uterine angiosarcoma, the median survival was reported to be 12.2 months (range 2-84 months) [10]. In cases of radiation-induced vaginal and vulvar angiosarcoma, survival times ranged from 7-30 months after diagnosis [11].

Surgery with R0 resection is the primary treatment goal for angiosarcoma [11]. As for systemic therapy, paclitaxel, anthracycline, and gemcitabine-based regimens are commonly recommended [12]. The ANGIOTAX study of weekly paclitaxel for unresectable angiosarcoma showed a median time to progression of 4 months. In this case, paclitaxel was effective, with a response time of 16 months [13].

Early diagnosis is critical for the treatment of angiosarcoma. In this case, a transvaginal needle biopsy played an essential role in obtaining a definitive diagnosis. Furthermore, NGS analysis was successfully performed on the biopsy specimens. With advances in personalized cancer treatment, the demand for specimen collection for genomic profiling is rapidly increasing.

Intrapelvic lesions can be biopsied via transvaginal ultrasound-guided, transrectal ultrasound-guided, or percutaneous routes. Percutaneous biopsy of pelvic lesions is difficult due to the presence of various organs and large vessels, so it is suggested that CT-guided biopsy be performed [14]. In transvaginal and transrectal approaches, the shorter distance from the transducer to the target organ may result in better diagnostic outcomes as compared to the percutaneous route [15]. However, infection-related complications have been reported following the transrectal approach. While the transvaginal route is not completely aseptic, the necessity of prophylactic antibiotic administration remains controversial [15]. Therefore, in this case, we opted for a transvaginal-guided biopsy and administered antibiotics prophylactically, without any complications.

Lung needle biopsies have been reported to provide adequate DNA and RNA quality for NGS analysis, with feasibility rates of 86% in most cases [16]. In terms of biopsy needles, it has been shown that an 18-gauge needle yields 4.8 to 5.7 times more material than a 20-gauge needle, suggesting that an 18-gauge needle is more suitable for obtaining a larger sample [17]. In this case, a somatic *BRCA1* mutation was detected by NGS analysis in a specimen obtained from an 18-gauge needle biopsy, but the patient was unable to participate in the clinical trial due to interstitial pneumonia. NGS analysis can be a useful tool in guiding treatment decisions for radiation-induced angiosarcoma, which has limited treatment options.

The findings of this case report are limited by the inability to generalize the results to a broader population due to the unique nature of the case. In cases of inoperable tumors, a needle biopsy may be useful for the pathological diagnosis and NGS analysis.

## Conclusions

This case highlights the diagnostic and therapeutic challenges of radiation-induced gynecological angiosarcoma, an exceptionally rare complication following cervical cancer radiotherapy. Pelvic masses that develop after radiotherapy for gynecologic cancers should be considered as potential radiation-induced sarcomas. RIAS in the gynecological region, although often unresectable, are very rare, and NGS analysis is essential for the genetic background in which they occur and for their diagnosis and treatment. We emphasize the utility of a transvaginal needle biopsy as an effective diagnostic tool, particularly when coupled with NGS analysis for comprehensive genetic profiling.
